# From Shock to Shift–A Qualitative Analysis of Accounts in Mid-Career About Changes in the Career Path

**DOI:** 10.3389/fpsyg.2021.641248

**Published:** 2021-02-26

**Authors:** Irina Nalis, Bettina Kubicek, Christian Korunka

**Affiliations:** ^1^Faculty of Psychology, Institute of Applied Psychology: Work, Economy, Society, University of Vienna, Vienna, Austria; ^2^Faculty of Natural Sciences, Institute of Psychology, University of Graz, Graz, Austria

**Keywords:** career shocks, career change, job change, mid-career, qualitative interview study

## Abstract

Career shocks are the norm, not the exception. Yet, much of research and counseling on career-development holds unrealistic assumptions of a makeable career. Little is understood about the role of shocks on the career path and how the interplay of individual reactions to shocks shapes careers. The purpose of this study is to provide understanding of responses to different attributes of career shocks and career shocks as antecedents to career and job change. A qualitative approach was chosen and data were obtained from 25 semi-structured interviews with a sample of mid-career individuals who had experienced shocks in their work lives. The analysis was 2-fold and aimed at unearthing of individual responses to shocks and the question of the role of shocks on changes in the career path. Firstly, the analysis of career shocks revealed a pattern of distinct agentic responses in relation to shocks of different attributes. Secondly, from the analysis of shock attributes and corresponding responses over time career changer profiles emerged which differ in regard to career change behavior and magnitude of changes in the career (e.g., major career changes into another field). A process model which depicts how post-shock careers are shaped distinctively in relation to different shock attributes and corresponding responses is presented. This study underlines the importance of understanding the unplannable in career development and shows a variety of options for individuals to develop their careers despite shocks. Limitation stems from the investigation of a sample limited to mid-career individuals. The findings provide a new conceptual lens to theorize and conduct research on career shocks and career changes and facilitate the development of coping strategies for career shocks. The originality lies in the investigation of the momentum of career shocks on career paths with detail to different attributes of career shocks and how they impact the career path.

## Introduction

Contemporary careers are increasingly complex. Unpredictable events stemming from changes both inside and outside organizations are likely to impact a career path (Barley et al., 2017). Yet, until today, most research on career development has focused on individual preferences and perceptions of career development, with relatively little attention paid to external factors (Akkermans and Kubasch, 2017; Kostal and Wiernik, 2017). Recently, many scholars (Gunz et al., 2011; Akkermans et al., 2018) have challenged this view of individual agency and criticized the ideal of a “makeable career” highlighting the impact of shocks on career development instead (e.g., Hirschi and Valero, 2017). According to Akkermans et al. (2018), the research on the interplay between agency and context is in decline since the rise of the new career paradigm. Therefore, the integration of perspectives such as the systems-theory framework on careers (McMahon, 2019) and its contribution on the understanding of the role of context or chaos theory of careers (Bright and Pryor, 2011) serve as a missing link in research on career development. Hence, this study follows the call to investigate the interplay “between career shocks and agency-related factors thereby building a bridge between both perspectives” (Akkermans et al., 2018: p. 8).

Following the critique of the “makeable career” and the demand for research on contextual factors, Akkermans et al. (2018) proposed a categorization of career shocks regarding five different attributes (frequency, predictability/controllability, valence, duration, and source). Shocks are defined as being caused by external factors and outside of individual control. Moreover, shocks can be perceived as positive (e.g., promotions) or negative (e.g., health problems). Additionally, responses to shocks may vary greatly according to individual resources to address challenges. Negative career shocks in particular require career resilience and adaptability (Johnston, 2018). However, research on new careers (Kuron et al., 2016), career self-efficacy beliefs (Betz, 2007; Kim and Lee, 2018), and the role of chance events on career decision-making (Bright et al., 2005) has found that attribution styles can buffer the negative aspects of experiencing shocks. Nevertheless, shocks can also be perceived as “a blessing in disguise” (Zikic and Klehe, 2006) and may account for positive outcomes (Holtom et al., 2012; Feng et al., 2020).

Within their conceptualization of different career shock attributes, Akkermans et al. (2018) refer to research by Seibert et al. (2016) in regard to potential of shocks to shocks trigger deliberate reflection and action pertaining to the career path.

According to Seibert et al. (2016), career shocks require an individual to rebalance career goals and consequently develop new paths to reach a goal. For instance, a shock could trigger an individual to act upon a desire for change in the professional life. Earlier studies on the relationship of shocks and turnover (e.g., Morrell et al., 2004; Holtom et al., 2005) showed how shocks lead individuals to consider current plans, and that shocks often precede changes.

Although changes in careers have long been the norm rather than the exception (Ahn et al., 2017; Barley et al., 2017; Kim et al., 2019), insight is needed on the interplay of external and internal factors leading to these shifts. In regard to changes of jobs or even major career changes to a different field, a shock might serve as a launchpad and prompt action in career paths that would otherwise be characterized by inertia (Lee and Mitchell, 1994; Morrell et al., 2004). Hence, this study aims to unearth the interplay of career shocks and changes on the career path.

This paper also responds to calls for more research on the impact of career shocks that integrates the time-sensitive perspective. For instance, Rummel et al. (2019) claim, it can be difficult to isolate shocks from their impact. Moreover, several authors claim that not all shocks precede changes nor lead to immediate action (Holtom et al., 2012; Akkermans et al., 2018; Rummel et al., 2019). Yet, due to the prevailing practice of studying careers at a single point, mostly at career entry levels, a better understanding of consequences of shocks over time is missing (Akkermans et al., 2018, 2020). However, research in mid-career offers underexplored venues for understanding the challenges and changes of career development (e.g., Ibarra, 2004; Van der Horst and Klehe, 2019). Moreover, investigation of mid-career individuals, allows to study career development over time.

## Theory

### Career Shocks

Career shocks are generally understood as unexpected events that occur outside the control of the individual. They further argue that the majority of the working population experience career shocks. Differentiation of shocks is provided through the newly developed categorization by Akkermans et al. (2018), who defined five attributes of career shocks: frequency, predictability/controllability, valence, duration, and source.

*Frequency*, as one distinct quality of a career shock, differentiates how often individuals experience shocks. Some shocks (e.g., being sexually harassed at work) occur more frequently than others (e.g., an environmental disaster). A question of special interest regarding frequency is whether it leads to habituation that inhibits reflection and action, for instance, due to change fatigue (e.g., Zeitz et al., 2009), or, to the contrary, whether frequent experiences of shock might even enable resilience (Bimrose and Hearne, 2012; Lyons et al., 2015). For instance, Akkermans et al. (2018) refer to life-satisfaction studies which have shown that recurring challenges in the domain of work (e.g., repeated phases of unemployment) tend to lead to heightened sensibility, whereas problems in private life (e.g., repeated divorce) stimulate habituation. Given these ambiguous results, it is important to contribute to the understanding of how the experience of multiple shocks impacts a career path over a longer period.

As another set of distinct shock attributes, Akkermans et al. (2018) differentiate *predictability* and *controllability*. Shocks have varying degrees of likelihood and controllability. Moreover, even planned events might cause unexpected consequences. For instance, even a planned pregnancy might nevertheless cause a career shock. On the other hand, employees can be informed about a layoff in advance, hence the shock is predictable to some extent, yet the job loss is not controllable. One strategy to address the predictability/controllability of shocks can be found in the attribution style of a growth-oriented mindset (Seibert et al., 2016). This growth orientation allows an individual to overcome restricting ideas about personal abilities and grow into a new role. To illustrate, a recent exploration of career adaptability of refugees showed how decidedly ignoring what is out of one's control while taking responsibility for what is within one's influence helps to regain control (Wehrle et al., 2019). This mindset could be also helpful in coping with career shocks.

*Valence* of career shocks refers to the evaluation of the shock's outcome. According to Akkermans et al. (2018), shocks that are positively valenced are assumed to have a positive impact on someone's career, with negative shocks leading to negative outcomes. A study by Blokker et al. (2019) in young employees showed the effect of positive career shocks on efforts to attain career success and employability, with negative career shocks having inverse effects. In contrast, Rummel et al. (2019), cite studies that found positive outcomes from negative shocks. The present study proposes one avenue to understand these contradicting results by disentangling the subjective components of the description of the shock experience and consequences, particularly with regard to the values individuals express. As Seibert et al. (2013) showed, shocks trigger deliberate reflection and action pertaining to the career path that could possibly lead to a general shift in individual career paths. Hence, this study assumes that reflection might also provoke questions of individual values, especially in cases of values violation (e.g., Hall, 2004). This relates also to the literature on broken psychological contracts (e.g., Rousseau, 1989; Clinton and Guest, 2014), and subjective success (Dries, 2011; Mayrhofer et al., 2016; Shockley et al., 2016). Moreover, Akkermans et al. (2018) suggest referring to Beach's (1990) image theory to include the idea of values and ideals when researching the impact of shocks on career development. Therefore, this study extends the view on the attribute of valence from a description of negative or positive shocks as described by Akkermans et al. (2018) to expressions of personal values and beliefs.

Another attribute of shocks is the *duration*. Duration can be experienced relative to the length of the shock event itself and the proximal or distal consequences. Furthermore, it is assumed that the impact of the duration of a shock differs with the length of the shock, in that a longer shock might show more severe consequences (Akkermans et al., 2018). Additionally, Lee et al. (1999) showed that a longer period of discomfort with the current position or while a person thinks about other career perspectives could interact with an unpredicted job offer and elicit change. It can be assumed that the stifling of career advancement over an extended period might kindle career change intentions and, especially. These intentions might be linked to self-concept and, breaches with the trajectory image, which according to image theory, describes the image a person aspires to achieve (Akkermans et al., 2018). Therefore, this study specifies the duration of shock experiences also in relation to barriers to individual career aspirations over time.

Finally, Akkermans et al. (2018) describe *source* as an attribute for shocks. They characterize sources as interpersonal, family-related, organizational, environmental, or geopolitical. Sources of shocks can also be assumed to concern questions of structural barriers, inequality, and injustice. To illustrate, research on the psychology of working theory (Duffy et al., 2016; Blustein et al., 2019) highlights that ethnic background and social class pose structural barriers and risks to career development. What makes the source of a shock also relevant for further career development is that hence source can be the most challenging attribute of a shock to overcome. Hence, it can be assumed that certain demographic characteristics increase the vulnerability to experience shocks. However, the source of shock holds potential to buffer against negative effects as it might trigger reflection and beneficial attribution styles (Seibert et al., 2013). For instance, if an individual has reasons to assume that the shock of job loss was not caused by personal failure, but has an external source, for instance mass layoffs, the source of the shock can act as a buffer (Bright et al., 2009). According to systems theory framework of career development (McMahon, 2019) the interaction between sources and individual action is shaped through an ongoing process of meaning making and individual agency. This process involves the knowledge about self and the environment. Hence, it can be assumed that, structural barriers need to be acknowledged, as well as the potential of meaning making in analysis of questions related to the source of shocks.

### Career Change

Across the globe, career changes have become a normality (Rice, 2014; Akkermans and Kubasch, 2017; Barley et al., 2017). For instance, data from the U.S. labor market show that people experience an average of 12 job changes throughout their careers (U.S. Bureau of Labor Statistics, 2015, cited in Ahn et al., 2017). Accordingly, career change literature captures the internal challenges and strategies to deal with career changes. Career changes entail monetary and psychological costs (e.g., Holtom et al., 2005), movement capital (Peeters et al., 2020), a large degree of adaptation (Brown et al., 2012; Johnston, 2018), and reinvention of work identity (Ibarra and Petriglieri, 2010).

In regard to the definition of what constitutes a career change and what differentiates it from a job change, Carless and Arnup (2011) distinguish between small and major career changes. Hence, their definition of career change is reduced to changes of the professional field which are largely unrelated to former experiences. This study extends this definition by building on the conceptualization by Ibarra (2004) who offers a definition of career change that entails any role change. Therefore, changes to a different field, a change of employer, or a change toward self-employment are included. Additionally, Lee et al. (1999) suggest that it is worthwhile to include non-work options (e.g., graduate school) or changes to entrepreneurship, as there are similarities in the search and evaluation process the individual has to perform. All types of changes on the career path are relevant for this analysis, as each type can be preceded by career shocks. Moreover, a broader definition of career change enables acknowledgment of the varying costs associated with the different types of changes (e.g., Holtom et al., 2005). Hence within this study, career change is considered on a spectrum from small adjustments within one's career path (e.g., changes in employer or position within the same field) to major changes of fields (e.g., retail to finance) and sector (e.g., changes from the public to the private sector). Moreover, changes of role (e.g., self-employment) are also covered. Small changes are referred to in this study as job change. Other changes are referred to as major changes.

To conclude, the purpose of this study is to clarify how career shocks ignite changes in the career and how the interplay of external shock experiences with internal career change responses impact the career path over a longer period. Given the relative neglect of research on shocks in the last decades and the novelty of the systemization of shocks by Akkermans et al. (2018), this study provides original insights on the impact of different characteristics of shocks on the career path.

The research questions are:

RQ1: **How do individuals respond to career shocks of different attributes?**

RQ2: **How does the impact of the interplay of different attributes of career shocks and responses shape the career path over time?**

## Method

### Research Design

This study applied a qualitative, explorative research method. A sample of 28 individuals, who had experienced shocks in their work biography, was recruited via purposeful sampling (Suri, 2011). For the purpose to select individuals with careers that had already developed over a certain period of time, mid-career individuals aged between 35 and 45 years were recruited. A semi-structured interview enabled the understanding of how career shocks ignite career change and impact the career path over a longer course of time. Inductive and deductive and analytical steps were combined for analysis. The aim was to unearth the role of career shocks in the phenomenon of changes in the career path (job changes, career changes).

In the following section the Gioia methodology for qualitative research (Gioia et al., 2012; Gehman et al., 2018) is outlined with special attention to the combination of data-driven and theory-driven analysis. Moreover, a step-by-step description of the coding process and illustration of the collaboration between co-authors is provided. Hence, each step of the analysis which led from the first-order analysis via second-order analysis to the development of the process model is explained.

### Recruitment and Participants

A sample of 28 individuals, who met the criteria of shocks in their work biography was recruited via purposeful sampling (Suri, 2011). Snowball sampling (Suri, 2011) allowed to obtain a diverse sample (education, ethnicity, gender, professions, sector, growing up in urban or rural areas). To specify, the central criteria for selection was the experience of ruptures in the career path. It needs to be highlighted, that the term rupture was used for recruitment instead of shock, in order not to superimpose the terminology by Akkermans et al. (2018) on participants. The second central sampling criterium was age, with the aim to explore careers that already developed over a certain period of time. Examination at mid-career allows greater insight into evolving career paths as the entry phase has passed, yet career exit is still years ahead. At the time of the data collection, participants had a minimum of 10 years of full-time work experience and were still at least 20 years from the official retirement age. To summarize, recruiting followed the criteria for ruptures in the work life and the mid-career stage of the work life, hence demographical criteria were not applied. However, the sample also allows for investigation of structural barriers to career development (e.g., in regard to gender, ethnicity or social class) as the sample was heterogenous.

First, an initial set of persons from the authors' network was contacted via email. Subsequently, snowball sampling (Suri, 2011) allowed reaching out to a diverse network where individuals with different backgrounds (education, ethnicity, gender, professions, sector, growing up in urban or rural areas) could be met. For recruitment, participation in a career biographic interview study of career paths with ruptures was called for. In regard to sample size, an original set of 28 interviews were conducted. The sample size follows recommendation by Guest et al. (2006), who suggest that the number of interviews follows agreement of the authors that further interviews would not generate new insights. However, three interviews had to be excluded for further analysis as these participants had answered the call for ruptures in the work life, but throughout the interviews it became visible that their careers were untouched by shocks and had remained unchanged.

The average age of participants at the time of the interview was 38.9 years. All participants were working (either employed or self-employed). Out of the final sample of 25 respondents, 13 were male, 12 female. All but one of the participants lived in a large European city; however, not all were originally from this city but also came from other countries or rural backgrounds. All but one participant were first language German speakers. Table 1 shows participant characteristics, the shocks they experienced, and related career changes. Within the table the spectrum of career changes applied by this studied is marked to distinguish whether the career changes were to another field, to another employer or to another role (including education or self-employment).

**Table 1:** Respondents characteristics.

**R**	**Sex**	**Age**	**Shock(s)**	**Career change(s)**
1	M	38	Goal Conflicts Job Loss	Nurse to Hair Dresser^*^ Barber to Self-Employment^***^
2	F	35	Job Loss	Consulting to Journalism^*^
3	M	45	Job Loss	Politics to Social Sector^*^
4	M	43	Accident Job-Offer Goal Conflict	Mechanic to Office Clerk^*^ Clerk to Manager in Education Sector^*^^,^^***^ Education Sector to Tourism^*^^,^^***^
5	F	35	Earthquake Fraud	Media Advisor to Strategist in Advertising Agency^*^ Strategist to Graduate Education^***^
6	M	44	Ethnicity Related Injustice Job Offer Economic Recession Conflicts with the Team Job Offer	Lawyer to Language Teacher^*^ Language Teacher to Consultant^*^ Consultant to Bar Man^*^ Bar Man to Language Teacher^*^ Language Teacher to Consultant^*^^,^^***^
7	F	37	Job Offer	Entertainment to Social Sector^*^
8	M	38	Job Offer Economic Troubles	Medical Doctor to Radio Station Manager^*^ Entrepreneur in The Design/Event Field^*^^,^^***^
9	M	40	Pregnancy	Business Consultant to Entrepreneur in Retail^*^^,^^***^
10	M	35	Financial Troubles of the Employer	Change of Employer^**^
11	F	35	Conflicts w/ Organizational Culture	Law to Art^*^
12	M	36	Job Offer	From Consulting to Management^*^
13	F	41	Change in the management Job Offer	Journalism to Administration ^*^ Administration to Marketing in Social Sector^*^
14	F	35	Goal Conflict Severe Injury	Fashion Retail to Handcraft^*^ Employment after Self-Employment^***^
15	F	39	Gender-Related Injustice	Communication to Energy Sector^*^
16	M	41	Job Offer	Administration to Social Sector^*^
17	F	37	Goal Conflicts Bankruptcy of the Employer	From Researcher to Consultant^*^ Self-Employment^***^
18	M	42	Economic Recession Accident	Banking to Real Estate^*^ Change of Employer^**^
19	M	40	Goal Conflicts	Marketing to Social Sector^*^
20	F	44	Goal Conflicts	Gaming Company to Personal Development^*^
21	F	42	Change in Management	Self-Employment^***^
22	F	37	Conflicts w/Management	Dental medicine to psychological counselor^*^
23	M	44	Job Loss	Researcher in physics to intern in advertising^*^^,^^***^
24	M	35	Conflicts w/Management Conflicts w/Management	Journalism to Politics^*^ Politics to Digital Advertising^*^
25	F	40	Economic Recession	Advertising To Public Affairs^*^^,^^***^

* marks changes to another field

** to another employer

*** * to another role (incl. education or self-employment)*.

### Instrument and Procedure

A semi-structured interview guideline was designed to take account of the interplay of external shocks and individual responses. The purpose was to capture behaviors and reflections (Bimrose and Hearne, 2012), with a set of seven open-ended question related to the career path. The interview started with a general invitation to talk about how participant's professional life had evolved. This was followed by open questions that invited a detailed articulation of the steps and shocks that led to their current career. To illustrate, the following three questions were used to prompt accounts of the shocks and changes that were experienced: How did your career evolve over time? What types of career ruptures have you experienced in your career path? What barriers or drivers did you experience on your way? This approach was chosen to gather data to unearth how the shocks triggered thought processes and deliberative action (Seibert et al., 2013; Akkermans et al., 2018). The interview questions are in the Supplementary Material.

The interview guideline was piloted with one interview. No major revisions were necessary as the questions seemed easy to understand by the participant and adequate for the purpose. To maintain consistency, the first author conducted all interviews. All interviews were conducted in German and lasted between 45 and 90 min; the difference in length resulted largely from the number of reported career shocks and the impact the shocks had on their careers, hence the numbers of reported shocks and changes. All interviews were audio-recorded and transcribed. Quotes have been translated from German to English by the first author, who is proficient in English, with back translation by the co-authors, who are also proficient in English. The anonymous and voluntary nature of the interviews was explained to the participants, who gave written informed consent prior to the interviews. Anonymity was ensured by labeling of the participants with a capitalized R and consecutive numbers (R1, R2, etc.). Participants received no compensation.

### Data Analysis

To analyze the qualitative data, we combined the inductive methodology of Gioia et al. (2012) with a deductive approach based on the five career-shock attributes of Akkermans et al. (2018). The methodology by Gioia et al. (2012) stems from grounded theory, and focuses on informant-centric terms yet also foresees the integration of theory-driven codes in the development of a process model. The computer software ATLAS 8.2.3 supported the analysis.

In line with Gioia et al. (2012), first-order analysis focused on informant-centric, original terms and codes of participants. In order to make the participant's voices heard, the analysis was primarily built on informant-centric terms and emerging data were allowed to shape the methodological process. Moreover, participants were seen as “knowledgeable agents” (Gioia et al., 2012); original verbatim is provided throughout the findings section.

The subsequent second-order researcher-centric analysis permitted to develop concepts informed by theory. The five characteristics of shocks as defined by Akkermans et al. (2018) served in this part of the analytical procedure as theory-based code which was combined with the informant-centric terms. Through stepwise comparing and contrasting of the individual career narratives and the detailed accounts of shocks and changes, a relation of specific responses to shock attributes as described by Akkermans et al. (2018) became apparent.

Research question one aimed at identifying responses to career shocks. In the first step, interviews were screened for accounts of shocks and changes independently. Open coding resulted in a very rough list of first-order codes for responses to shocks of different attributes (e.g., again new as expression for frequent experiences of shocks). The first-order codes where then elevated by theory to second-order themes. Second-order themes allow comprehension of the deeper structure of the concept, for instance in regard to frequent experiences of shocks, accounts of habituation and resilience were found. The next step aimed at identifying aggregated dimensions (Gioia et al., 2012). These aggregated dimensions stem from emerging concepts that explain the phenomenon of responses related to career shocks. To illustrate, “change muscle” was identified as the aggregate dimension of the response to the career shock attribute frequency.

In order to answer research question two, which aimed at the understanding of the impact of the interplay of career shocks and responses on the career path over time, a new round of individual analysis of each career narrative was performed. Therefore, the transcripts were reviewed to explore how within each career narrative the responses to career shocks of different attributes might have shaped distinct profiles. From this analysis, career changer profiles emerged from what was found to be a dominant interplay of career shock and change responses. Analogous to the steps in the analysis for research question one, the career changer profiles were also distilled from first-order code, to second-order themes and lastly resulted in aggregated dimensions. It is important to note that career changer profiles become visible only from the perspective of a longer course of time. Similarly, profiles can only be identified by contrasting and comparing the impact of the interplay of shocks and responses throughout analysis of more than one instance of shock and change within one career narrative and with other accounts. To illustrate, career paths that were predominantly shaped by the interplay of shocks of the attribute of valence and the corresponding response showed in the profile of a maverick and resulted more often in major career change. In contrast, paths that were mainly impacted by the interplay of shocks attributed to duration and their corresponding responses.

### Findings

Participants of this study reported career shocks, listed in alphabetical order, in regard to accidents, bankruptcy of the employer, changes in the management, conflict with the organizational culture, conflicts with the management, conflicts with the team, earthquake, the economic recession after the financial crisis, financial troubles of the employing company, fraud in the employing company, gender- and ethnicity-related injustice, goal conflicts, job loss, job offer, unplanned pregnancy, and severe injury. This list of shock resulted from the first round of coding and were subsequently categorized according to career-shock attribute by Akkermans et al. (2018). In the following the findings are grouped career shocks of different attributes and the responses which they ignited; the second part is dedicated to career changer profiles which emerged from the analysis of post-shock careers and how they were shaped by the interplay of career shocks and responses.

**Figure 1:**
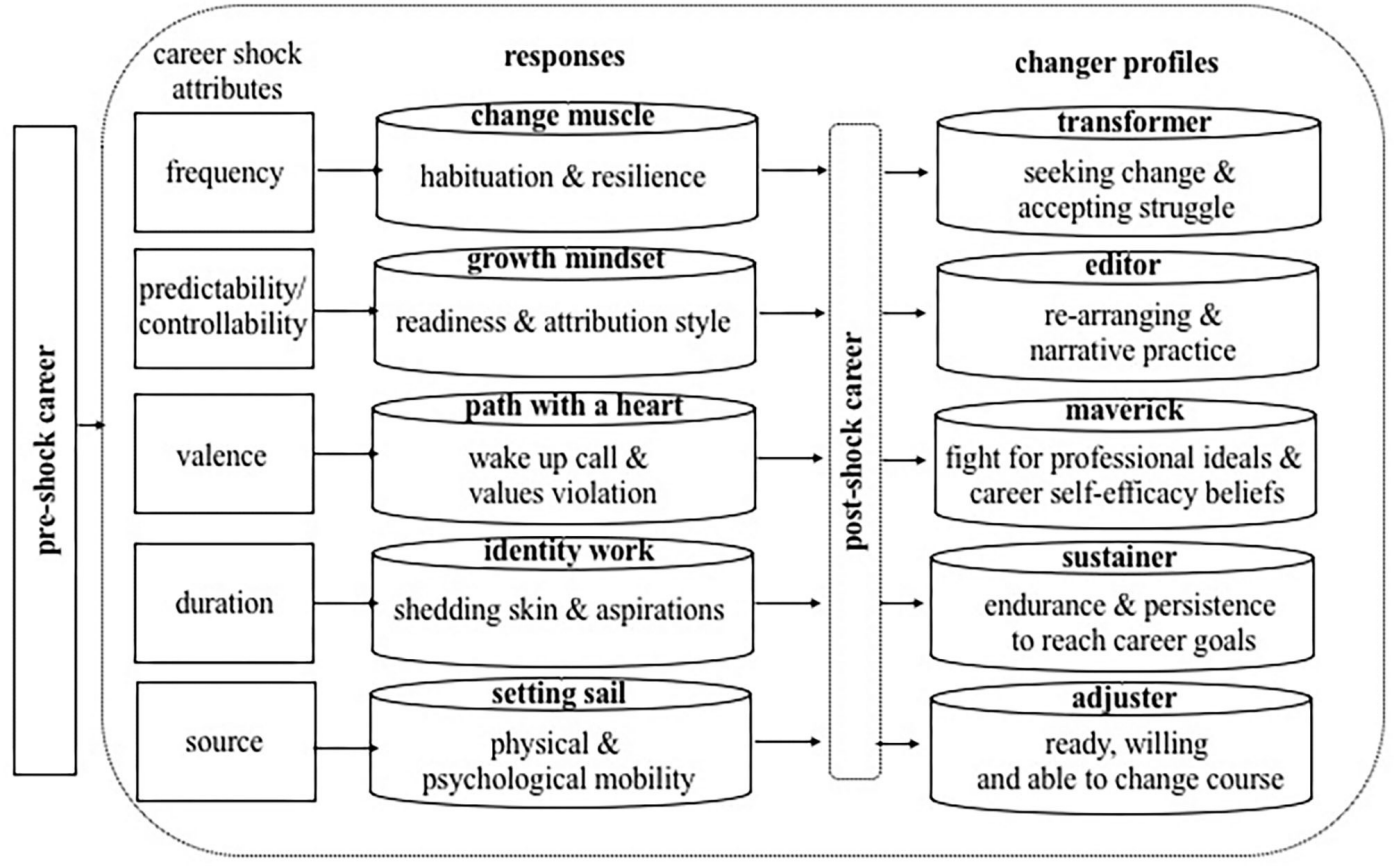
Process model from shock to shift. The figure presents aggregated dimensions of responses and career changer profiles.

In the following the findings are grouped by career-shock attribute according to the systematization by Akkermans et al. (2018) with the first section dedicated to career shock responses; and the second part to career changer profiles which emerged from the interplay of career shocks and responses over time. A graphical summary of the findings to research questions 1 and 2 is presented in Figure 1. Notably, Figure 1 shows the process from shock to shift via aggregated responses to career shocks and the post shock career which showed in distinct career changer profiles related to each shock attribute.

#### Response to Frequency: Change Muscle

*Frequency*, as one distinct quality of a career shock, differentiates how often individuals experience shocks. Some shocks (e.g., being sexually harassed at work) occur more frequently than others (e.g., an environmental disaster). A question of special interest regarding frequency is whether it leads to habituation that inhibits reflection and action, for instance, due to change fatigue (e.g., Zeitz et al., 2009), or, to the contrary, whether frequent experiences of shock might even enable resilience (Bimrose and Hearne, 2012; Lyons et al., 2015). For instance, Akkermans et al. (2018) refer to life-satisfaction studies which have shown that recurring challenges in the domain of work (e.g., repeated phases of unemployment) tend to lead to heightened sensibility, whereas problems in private life (e.g., repeated divorce) lead to habituation. Given these ambiguous results, it is important to contribute to the understanding of how the experience of multiple shocks impacts a career path over a longer period.

In this study, accounts of frequent ruptures and surprising turns were used by the participants to illustrate how change and shock became routine on their ever-changing career paths. Habituation to multiple experiences of shocks was reported, which appeared to serve as a facilitator of change and stimulated increase in resilience. Participants in this study seemed to have gained strength that helped them re-shape their careers according to their needs and interests. The experience of frequent shocks was found to be associated with habituation that led to the development of a *change muscle*. However, frequent experience of shocks rarely triggered immediate career change it rather appeared to serve as a mindset for coping with shocks. This is highlighted by these two participants, who voiced how they perceived change and shock as routine on an ever-changing career path.

My previous professional life was actually characterized by constant learning and constant change of sector

– this was incredibly important. (R18)

Many, many lessons on how things change. And I think that has shaped me anyway, … tomorrow it can always be different. You get up and get fired and that's it. (R6)

#### Response to Predictability/Controllability: Growth Mindset

One strategy to address the predictability/controllability of shocks can be found in a growth-oriented mindset (Seibert et al., 2016), which allows an individual to overcome restricting ideas about personal abilities and to grow into a new role. This was mirrored in several interviews, as individuals who predominantly reported shocks with the attributes of predictability and controllability responded with the development of a *growth mindset*. Positive attribution styles and the ability to adapt readily were apparent from the interviews. This is illustrated in the statement of a career changer, who had an accident early in his career as a mechanic which resulted in a retraining that brought him into the educational sector, where soon after entering he was offered a leadership position first on the local and then on the national level:

Then it also happened by accident that the country manager was leaving and asked me if I wanted to do it in his place and I was ready to change anyway. (R4)

Another respondent, who actually had just finished his medical studies got an unexpected job offer in an entirely different sector, which he accepted nevertheless:

And then they asked if I wanted to join this new radio station. (R8)

For participants in this study, the experience of unpredictable and uncontrollable shocks did not cause resignation but stimulated action. What also showed in the data, many of the reported accounts of unpredictable events were in relation to surprise job offers. It can be assumed that the growth mindset applied, did equip participants with the required emotional regulation and resilience to accept these offers.

#### Response to Valence: Wake-up Call

How shocks were valenced showed a strong link with a more general question of values and turned out to have a strong impact on participants' careers. What was initially a shock served for some participants as a *wake-up call* that encouraged the pursuit of new endeavors. Experiences of values violation altered the career paths substantially—most participants did not continue in the field where they had encountered this type of career shocks, as shown in the case of a former nurse who changed to become a hair dresser:

The medical system in (city) is anyway … you get exploited and you get no supervision …I could not bear it any longer …I told myself ok I have to get out of there somehow. (R1)

After the change to become a hair dresser, he also experienced a job loss, which led him to remain a hair dresser, but leave his role as employee:

I'm not angry, that gave me a good boost. It's a huge step to becoming self-employed. (R1)

Another respondent reported the shock of a job loss as a trigger for a major career change from business consulting to journalism:

I already had started to think that I still want to be a journalist and that that was always my heart's desire and then happened one of the dirtiest stories of my professional life. We got an award and were on the podium and blah and (were told that) we are so excellent and 4 months later I was fired. (R2)

Additionally, a distancing and inner movement away from organizational goals was found.

We as a team chose to rather let the project go down, than to surrender to these company norms. (R24)

#### Response to Duration: Identity Work

According to Akkermans et al. (2018), the attribute of duration, needs to be regarded from two perspectives: the duration of the shock itself (e.g., the recovery from an injury usually takes longer than the termination of an employment contract) and the consequences of a shock. Respondents who narrated shocks in regard to duration, highlighted demands to develop their vocational identity; they either fought to maintain their identity and career aspirations despite shocks or had to undergo an intense phase of emotional but often also financially taxing change of role. An illustration of both facets of duration is shown in the case of a craftswoman who had suffered the shock of an injury and felt very insecure about her professional future for a sustained period:

That was very hard, because I did not know how well I'll ever be able to stand in an upright position again in my life and if I can ever continue such a job. That was the really big question and of course my employer was wondering, too. (R14).

Adding to the shock experience of the injury, at the time of the incident she was self-employed and lacked the security of being able to maintain her position in one of the rare outlets where she could perform her craft on a professional level. Eventually she endured in order to maintain her dream profession.

I waited for years to become employed. (R14)

This transition process also prompted reflection on identity questions that showed in a procedure alike to shedding skin and renewal from one self-image to a new self:

Those were the phases in my change. The first phase was the total rejection of everything that had to do with my original job. (R15)

#### Response to Career Source Related Shocks: Setting Sail

Shocks of the type of source were experienced especially strong as a structural force on the individual career path. Within the interviews the source of a shock was shown in accounts of discrimination. Moreover, the shocks were clearly framed as barriers. What surfaced prominently within this study were reports of sexism and racism.

I had the feeling he just wants to break me *per se* because I'm a woman. That I don't have a say in here, as it is just a very male dominated company. I think 60, 70% only men and the women actually all held assistant positions, only. (R2)

You did not see from the CV that I was brown-skinned. At a job interview where I wanted to be … I was out already when I walked through the door. As a consequence, I did not get the job. (R6)

Within the interviews, the source of a shock was mentioned in several accounts of discrimination related to ethnicity or gender but also in relation to shocks in the private realm. In both matters, most clearly voiced responses were linked to *setting sail*, that is, physical or psychological mobility. Shocks that were caused not by structural injustices but were related, for instance, to broad economic turbulence affecting the entire industry, often triggered a departure from what was described as a harmful environment. Responses were narrated either as adaption behavior or through perspective taking. What followed these shocks was often described as a move to new lands, either physically or mentally. Most responses were thus linked to leaving either the place or job.

We got to know each other and she got pregnant relatively soon afterwards and it was never a question for her to move to Austria. She said you can come to Indonesia, if you want to see your kids. (R9)

This respondent left the job environment to pursue a career change to graduate education:

I saw the ship sinking … I have to get out of there and then I saw the managing director escorted out by bodyguards and then some shareholders from Canada came with their trolleys and kicked people out one by one; and then I said well … I am going to take an educational leave. (R5)

### Career Changer Profiles

The findings to research question 2 describe how the interplay of certain shock attributes and corresponding responses shaped the career path. The analysis showed different attributes of shocks ignite distinct responses which eventually shape career paths toward distinct career changer profiles. It became apparent, that the impact of the interplay of shocks and responses, differed according to the shock attribute. Albeit individuals might have experienced the interplay of various shock attributes and change responses, the analysis revealed profiles that were predominantly shaped from the interplay of shocks and corresponding responses of a certain attribute over time. The identified career changer profiles provide insight into post-shock career self-management strategies.

#### Career-Changer Profile Frequency: Transformer

Career-changers of this profile show a tendency to embrace change and accept struggle. Individuals took on an attitude of a transformer of their work life, in which they often changed careers or jobs by seeking changes and accepting the struggles they met on their career paths quite voluntarily. Within the data for this study, the interplay of frequent career shocks and responses, resulted mainly in major career changes which involved changes of sector, field, occupational status (e.g., from employee to entrepreneur) and also retraining.

Examples include the story of a mechanic who became an academically educated manager in the educational sector and then an entrepreneur in tourism. His career path included many changes that were consequences of shocks: first, as a mechanic he had an accident that led to retraining as a sales clerk. From this position, he got an unexpected job offer to work in the educational sector, where he soon got promoted to manager. After several successful years in this position, he received another unexpected job offer as the country manager for the organization he was already working with, which involved moving to another city. However, after a few years in this leading position, he felt burdened by the routine, which triggered starting postgraduate education. After completing the master's degree, he did not return to his former position, but start change his career path toward entrepreneurship in tourism.

#### Career-Changer Profile Predictability/Controllability: Editor

Participants with experiences of either unpredictable or uncontrollable situations showed a tendency to accept the situation and to develop a rather pragmatic outlook on their work lives. Correspondingly, the changes described were smaller adjustments, yet within the same field and professional role. The term “editors” was developed for career changers whose stories were mainly affected by shocks of the typology predictability/controllability. Although their career path was not as originally intended, and thus they had to depart from the ideal of being the director of their personal career story, they used the material they were presented with and found ways to make it their story anyway. Hence, such career changers are like a movie editor who neither writes the script nor directs the shooting of the film, but through cutting and rearranging is able to make a consistent story out of the otherwise possibly disorderly material without rewriting it. Career changers of this type adjusted to a situation and changed the circumstances of their careers, instead of leaving to an entirely new field. However, the responses to career shocks were not about resignation but about active shaping of the situation to maintain their current careers.

Within this study, several stories were told of taking up graduate education and speculative applications for new jobs as preventive measures to buffer future shocks. An example of this profile is described in the case of a marketing professional who urgently wanted to change from the private to the public sector. However, due to lack of experience she did not secure a position for an extended period. The change occurred only after the positive shock of an unpredictable job offer, where she was introduced to the marketing department of a large social enterprise.

#### Career-Changer Profile Valence: Maverick

Shocks related to valence, especially if values violation was experienced, were shown in profiles involving major changes from one sector to another. Accounts also demonstrated aspects of struggles with hierarchies and supervisors, especially once participants started to act upon their professional ideals (e.g., regarding questions of justice in the workplace). Moreover, former ideas of a possible future-self (such as having studied journalism as a means to fight inequality but actually working as a business consultant) were reignited. A clear tendency toward strengthening career self-efficacy beliefs was also visible among career-changers of this profile.

Examples include the story of a business consultant who was a journalist by training and eventually became an investigative journalist after leaving the well-paid consulting world behind. Another account was found of an assistant in a law firm became a management director in the field of arts. Both profiles showed, that valence related shocks triggered not only to leave their former professional networks but also recalibrated their personal goals and ideas of success toward more subjective goals than money or status.

#### Career-Changer Profile Duration: Sustainer

Akkermans et al. (2018) argue, that shocks that are longer in duration have more severe consequences. The duration of a career shock was used by several participants as descriptor for either their persistence to stay within the current position or the perseverance to obtain for a new professional role. Career narratives that held accounts of duration, were termed *sustainers*. However, as the term suggest, duration in itself did not ignite career changes. Duration was rather described as an experience in the background of the transition process which was necessary to grow into the new role.

The length of the shock triggered in several participants an attitude of endurance, especially if the shock was related to a career they actually wanted to maintain. An example is shown in the shock of a lay off one career changer experienced after he left his job as a scientist over the age of 35 to start anew in an advertising company. However, he had problems adapting to the speed of his new work environment, which first led to a dismissal which was eventually retracted. Eventually, he could prove his talent, which led to some of the highest awards possible in the field.

#### Career Changer Profile Source: Adjuster

The interplay of the duration of the shocks and individual responses, had similar to duration, little distinctive aspects that led to career change. Therefore, more job changes than career change surfaced from analysis of career paths that were predominantly shaped by shocks of the attribute of source and their corresponding responses. It seems that sources prompted adaptation processes, which involved action and reflection. Thus, this profile was termed *adjuster*, which was signified by the adaptation of the personal view of the job, rather than a change of the career itself.

For instance, family needs that required moving to another country did not necessarily prompt questioning of the career but did require searching for a new field of work because the former job was not available in the new country. It was also shown that shocks caused by organizational troubles (e.g., mass layoffs) that could therefore be attributed entirely to external factors hindered deeper reflection. Although setting sail was the reaction and showed in physical and psychological mobility, this profile was characterized by a pragmatic stance.

## Discussion

### Main Findings

The purpose of this study was to clarify how career shocks impact career paths and ignite career changes. Earlier research has shown that shocks throughout the work life are common and that they impact long-term career outcomes (Bright et al., 2009; Akkermans et al., 2018). Hence, it follows claims to provide a complementary view on career development (Akkermans et al., 2018), that bridges context, herein examined from the perspective of shocks, and agency, which was found in individual responses and career changer profiles. To summarize, the findings show individual responses to different attributes of career shocks and career changer profiles that emerged from the interplay of career shocks and responses over time.

The analysis unearthed the impact of shocks on career paths and found different responses to shocks according to how the shock was described. Thus, although several respondents reported a similar shock experience (e.g., job loss) this study revealed that certain shock attributes led to specific responses which eventually shaped the entire career path distinctly. To illustrate, if the description of job loss bore indicators of the shock attribute of e.g., valence in contrast to e.g., the shock attribute predictability, a different impact on the career path became visible. Furthermore, the post-shock career revealed a certain pattern overtime in that the interplay between specific shock attributes and responses resulted in distinct changer profiles. Individuals with different changer profiles seemed displayed distinct attitudes and behaviors in regard to their career development.

Another observation of this study challenges the view that not all shocks precede changes as within the data all shocks ignited change responses which sheds a new light on the questions on the voluntary nature of career changes. Moreover, career change literature does not emphasize the scope of career change but rather distinguishes whether the turnover is voluntary or involuntary (e.g., Feng et al., 2020). Therefore, the tendency to distinguish career changes from the perspective of voluntary or involuntary nature of career changes (e.g., Feng et al., 2020) might thus be expanded on the perspective of the process from pre- to post-shock career as derived from our findings.

Within this study it became visible that shocks of different attributes also showed in different magnitudes of career changes. One possible explanation lies in the different attributes of shocks, of which some triggered more reflection than others. Shocks which triggered more reflection than others were shown to lead to major career changes. This became most visibly in cases of shocks related to valence where career changes to different fields were stimulated. Once individuals sensed their values had been violated, they were triggered to think of their ideas and ideals of work, eventually steering them toward a “path with a heart” (Hall et al., 2018). The predictability/ controllability and frequency of shocks also appeared to ignite major career changes. Predictability/controllability of shocks and major career changes, might be linked to the growth-oriented mindset (Seibert et al., 2016) applied by individuals who experienced shocks of that attribute. Therefore, growth-orientation might serve as strategy to overcome restricting ideas about personal abilities and enables growing into a new role. Instead, shocks related to duration and source ignited change action, yet stimulated less reflection. Correspondingly, shocks with these characteristics seemed to lead to smaller adjustments (e.g., change of employer) in the career path. Finally, source-related shocks were often described as structural barriers (e.g., in relation to gender, ethnicity) and led to smaller changes in the career. However, questions of the source of the shock might also be closely related to available resources to deal with challenges. As career changes involve emotional as well as financial costs (Peeters et al., 2020, Holtom et al., 2005), the available resources to transform a shock into a desired career change, might vary greatly.

### Limitations, Theoretical, and Practical Implications

This study demonstrates that, whereas individuals cannot control the occurrence of shocks, they can respond to shocks in an agentic manner.

A limitation to these findings is the sample which came from a specific age group and life stage, as the investigation of career paths over time was central for this study. Moreover, it was purposefully sampled in regard to ruptures in the work life. Yet, a limitation in regard to the sample concerns the question of the educational level and how this might translate on the abilities to deal with shocks. The sampling was based on the criteria for ruptures in the work life and the mid-career stage of the work life, hence did not sample for demographical information. However, the educational level was high with most of the participants which also showed relation to the experience of career shocks. To illustrate, albeit some of the participants started their career paths as low-skilled workers, eventually they went through re-training or upgraded their education oftentimes as a response to a career shock. Therefore, we believe, that for the purpose of this study the sample do not limit the findings. Moreover, as Akkermans et al. (2018) argue that career shocks are experienced by the majority of the working population. Nevertheless, future research could try to establish understanding of the relation of educational background with responses to career shocks.

Another, limitation lies in the focus of this study on dominant patters of the interplay between shock attributes and response. Thus, the interaction with other shock characteristics on the career path cannot be excluded. For instance, in the analysis it became visible that shocks that were described in regard to their duration and resulted in persistence to could also be linked to questions of valence. Thus, it is possible that persistence was fueled by a value-orientation that helped to maintain a “path with a heart” (Hall et al., 2018, Hall, 2004). Moreover, as source related shocks were often mentioned in relation to structural barriers, they might interact strongly with other shock attributes, e.g., valence in regard to questions of justice.

With regard to theoretical implications, the process model from shock to shift aims to serve as a building block to develop propositions about the role of shocks on careers from responses to post-shock careers characterized by career changer profiles. Future research could build on this process model to examine outcomes of the interplay of shock and responses. Hence, propositions about the role of shocks and possible outcomes could be derived and empirically tested. Three central topics have been identified as potential avenues for future research on the interplay of shocks and changes on the career path: subjective success, employability and transferability of skills.

Regarding subjective success, the analysis showed the individual definition of success might be altered by shocks, especially in cases where career shocks of valence were reported. As shown in earlier research on broken psychological contracts (e.g., Clinton and Guest, 2014 on the role of psychological contract breaches and turnover), the notion of success shifted toward a more subjective definition. The literature on the protean career (e.g., Gubler et al., 2014; Hall et al., 2018) and on calling as a driver of career change (Ahn et al., 2017) could serve as basis for further investigation the relationship of career shocks and subjective success. Moreover, Akkermans et al. (2018) argue that the reaction to a shock can be a central determinant for future career success. Furthermore, newly developed scales for measuring subjective success (e.g., Mayrhofer et al., 2016; Shockley et al., 2016) could be applied in at least two directions: to examine whether career shocks alter the individual definition of success and if career changes can lead to subjective success.

In regard to employability, it will be necessary to link understanding of the development and outcomes of post-shock careers to the perspective of vulnerable populations. One central concern related to agency on career paths is, that it may only be valid for those who are already highly employable (Forrier et al., 2018). The findings showed that ethnic background (e.g., Owens et al., 2019) and social class (Duffy et al., 2016; Blustein et al., 2019) pose structural barriers and risks to career development. Further research could build on the findings to develop questionnaires on career changing behavior that allow for better combination with demographic data. For researchers who wish to engage in further qualitative investigations, it could be useful to modify a future interview process by requesting that participants provide a copy of their curriculum vitae prior to the interview (Bimrose and Hearne, 2012) and to integrate demographic data (e.g., age, income, marital status) in the analysis. Accordingly, research on career guidance in multicultural societies (Sultana, 2017), should be consulted for the development of interview guidelines and questionnaires.

A third avenue for future research, concerns the question of transferability of skills and their role in coping with shocks and changes in the career. Transferability seemed to be central to the respondents as they reported how they used skills acquired in one sector in their new job or even field. It can be assumed that through a process of sense-making in narrative practice (Ibarra and Barbulescu, 2010) can serve as a stabilizing force in the transition process from one job or field to another. Moreover, there might be a link between employability, transferability and frequency of shocks. From the findings, it appears that the experience of multiple career cycles is fueled by transferable skills that allow adequate responses to frequent career shocks. In addition, the level of education and the degree of specialization might also be linked to questions of transferability of skills.

From the practical perspective, this study provides insights and illustrations for the development of career counseling interventions findings for the development of career counseling interventions with a focus on challenges arising in post-shock careers. The findings underline that individuals need assistance in developing coping strategies to deal with frequent career shocks (Seibert et al., 2013, 2016). It is essential to aim for the assistance in the development of coping strategies.

For instance, interventions could focus on the various costs of turnover such as movement capital (Peeters et al., 2020), adaptation (Brown et al., 2012; Johnston, 2018), and reinvention of work identity (Ibarra and Petriglieri, 2010) and integrate the findings from this study to illustrate the process from shock to shift. To illustrate, with the use of the findings from this study illustrations and stimuli could be developed, that elucidate the nature of shocks and the options for agentic responses. Moreover, counseling needs to acknowledge challenges for vulnerable populations (e.g., Blustein et al., 2019).

Other applications could aim at increasing individual resources to respond to shocks from the backdrop of the responses and career changer profiles identified in this study. To illustrate, with the use of the findings from this study illustrations and stimuli could be developed, that elucidate the nature of shocks and the options for agentic responses. Moreover, by training of attribution styles (Seibert et al., 2013 of a growth-oriented mindset as shown in this study as response to frequent shocks) and in training of the “change muscle,” resources and of career self-efficacy beliefs (Betz, 2007; Kim and Lee, 2018), could be increased.

Additionally, contextualization of shocks could also help in destigmatizing the experience of shocks which could eventually also help in coping. Earlier research has shown that if the individual has reasons to assume that the source of the shock is not individual failure, it acts as a buffer against the effects of shocks (e.g., Bright et al., 2009). This is of particular relevance in counseling with respect to structural barriers to career development (Duffy et al., 2016; Blustein et al., 2019). Counseling that helps in contextualization of the shock while aiming at increasing the availability of agentic behavior could combine illustrations of post-shock careers derived from the career changer profiles described in this study with facts from general labor market statistics.

In regard of the potential of shocks to trigger reflection, career counseling could encourage reflection as a career competency (Akkermans and Kubasch, 2017). According to research on reframing (e.g., Nowlan et al., 2015), individual reflection can lead to a process of sense-making by deriving personally relevant meaning from experiences. Hence, it seems valuable to further strengthen the understanding of the role of individual career narratives and the role of reflection in the process from shock to shift. Moreover, reflection on shocks could also lead to personal reevaluation of success. Therefore, future career interventions could integrate the findings in regard to shocks related to valence with research on subjective careers success as overcoming- For instance, scales on subjective success by Mayrhofer et al., 2016; Shockley et al., 2016), could provide multidimensional measures of success.

Lastly, in addition to the target group of career counselors, practical implications of this study entail the organizational and societal perspective. Thus, organizations or governmental institutions resources should likewise aim to integrate understanding of the prevalence of shocks and individual responses. Practitioners in human resource development plans as well as policy makers should aim for providing resources to address challenges in the development of post-shock careers for instance by better availability for re-training.

As a final remark on practical implications, as this paper was completed during the COVID-19 pandemic, it aims to provide a perspective on how to deal with career shocks related to this global crisis that has severely affected the labor market, as outlined in recent publications by Akkermans et al. (2020) and Hite and McDonald (2020). Although the data for this study were collected before the pandemic, this paper might contribute perspectives on the interplay of shocks and individual options to overcome rough patches on career paths, whether they are caused by the coronavirus or other external factors. It can be assumed that much of the findings have the potential (e.g., working with the responses and profiles to identify individual coping strategies) to be applied in career counseling.

## Data Availability Statement

The raw data supporting the conclusions of this article will be made available by the authors, without undue reservation.

## Ethics Statement

The studies involving human participants were reviewed and approved by Internal Review Board, Faculty of Psychology, University of Vienna. The patients/participants provided their written informed consent to participate in this study. Written informed consent was obtained from the individual(s) for the publication of any potentially identifiable images or data included in this article.

## Author Contributions

IN conceived the main conceptual ideas. BK and CK supervised the design, sample, and planning of the interview study. IN carried out all the interviews. The coding process was performed collaboratively by IN, BK, and CK as a circular approach. All authors discussed the findings, the development of the process model, and contributed to the final manuscript.

## Conflict of Interest

The authors declare that the research was conducted in the absence of any commercial or financial relationships that could be construed as a potential conflict of interest.
