# Supplementary material for: From Shock to Shift–A Qualitative Analysis of Accounts in Mid-Career About Changes in the Career Path
Source: Front Psychol. 2021 Feb 26;12:641248. doi: 10.3389/fpsyg.2021.641248 (PMC7952510; doi:10.3389/fpsyg.2021.641248)
Supplement: Supplementary file 1 [file Data_Sheet_1.docx]

A1

APPENDIX

Interview guide line

1. How did your career evolve over time?
2. What types of career ruptures have you experienced in your career path?
3. What barriers or drivers did you experience on your way?
4. Please tell me more about your professional situation today.
5. What role did the people around you play in the development of your career?
6. Please tell me what is important to you at work.
7. How does your journey continue?
